# Wavefront Automated Refraction Comparison of Three Different IOLs: Aspheric Monofocal and Two Enhanced Monofocal IOLs

**DOI:** 10.3390/vision10010006

**Published:** 2026-01-26

**Authors:** Arthur Buffara van den Berg, Roberta Matschinske van den Berg, Bernardo Kaplan Moscovici, Maya Dodhia, Larissa Gouvea, Wallace Chamon, Karolinne Maia Rocha

**Affiliations:** 1 Department of Ophthalmology and Visual Sciences, Federal University of São Paulo (UNIFESP), São Paulo 04038-001, SP, Brazil; arthur-vdb@hotmail.com (A.B.v.d.B.); betamats@hotmail.com (R.M.v.d.B.); wchamon@pobox.com (W.C.); 2 Department of Ophthalmology, HO Londrina-Hospital De Olhos de Londrina, Londrina 86055-585, SP, Brazil; 3 Storm Eye Institute, Medical University of South Carolina, Charleston, SC 29425, USA; dodhia@musc.edu (M.D.); gouveaalarissa@gmail.com (L.G.); karolinnemaia@gmail.com (K.M.R.); 4 Department of Ophthalmology, Hospital Oftalmológico Visão Laser, Santos 11015-001, SP, Brazil

**Keywords:** cataract, cataract extraction, intraocular lenses, refraction, aberrometry, visual acuity

## Abstract

The objective of this study was to compare subjective manifest refraction with wavefront-based automated refraction using iTrace (ray tracing) and LadarWave (Hartmann–Shack) in eyes implanted with two enhanced monofocal intraocular lenses (IOLs) and a standard aspheric monofocal IOL, emphasizing agreement and refractive variability across optical designs. This retrospective cohort included 84 eyes from 42 patients implanted with Tecnis Eyhance (DIB00), RayOne EMV (RAO200E), or Tecnis ZCB00 IOLs. Postoperative evaluation (1–3 months) included uncorrected and corrected distance visual acuity and subjective manifest refraction, followed by automated refraction with iTrace and LadarWave. Outcomes were sphere, cylinder, and spherical equivalent (SE). Agreement was assessed using mean signed difference, mean absolute error, root mean square error, Bland–Altman limits of agreement, proportions within clinically relevant thresholds, and vector astigmatism (J0, J45). Linear mixed-effect modeling evaluated SE differences across methods and IOL types while accounting for within-subject correlation. Subjective SE differed among IOLs (*p* = 0.027), with RAO200E more myopic than ZCB00 (−0.20 ± 0.32 D vs. −0.08 ± 0.44 D, *p* = 0.035). Automated refraction showed greater variability and poorer agreement in enhanced monofocal IOLs, particularly for cylinder and SE, with wider limits of agreement and fewer eyes within ±0.50 D compared with ZCB00. In mixed-effect contrasts (three-method repeated-measures model), iTrace and LadarWave showed a consistent myopic bias versus manifest refraction in DIB00 and RAO200E, whereas in ZCB00 the iTrace–manifest difference was not significant and LadarWave retained a significant myopic bias. Enhanced monofocal IOLs exhibit reduced agreement between wavefront-based automated and subjective manifest refraction compared with a standard aspheric monofocal IOL. Manifest refraction remains essential for postoperative assessment, and automated measurements should be interpreted as complementary, particularly in IOL designs that modify aberrations.

## 1. Introduction

Cataract remains the leading cause of reversible blindness worldwide, accounting for more than 50% of global blindness according to the World Health Organization [1]. Advances in surgical technique and intraocular lens (IOL) design have transformed cataract surgery from a restorative to a refractive procedure, offering patients the potential for spectacle independence. Standard aspheric monofocal IOLs, such as the Tecnis ZCB00, provide excellent distance vision by incorporating negative spherical aberration to compensate for average corneal spherical aberration and improve optical quality [2,3]. However, their single focal point often necessitates spectacle use for intermediate or near tasks, limiting functional range [2,3].

Multifocal and trifocal IOLs were introduced to address these limitations by redistributing light into multiple focal points, thereby extending visual function at different distances. Several studies have demonstrated significant improvements in near and intermediate vision with these designs [4,5]. Nevertheless, disadvantages such as reduced contrast sensitivity and photic phenomena, including halos and glare, affect up to 10–15% of patients [6,7]. In some series, up to 5% of implanted multifocal IOLs required explantation due to dissatisfaction [8]. These drawbacks underscore the need for alternative solutions that can balance extended vision with fewer optical side effects.

Enhanced monofocal IOLs were developed as such a compromise. By inducing controlled amounts of higher-order aberrations, particularly positive spherical aberration, these lenses provide an extended depth of focus without splitting light [9,10]. The Tecnis Eyhance (DIB00, Johnson & Johnson Vision) incorporates a continuous aspheric anterior surface with increasing central power, thereby improving intermediate vision while maintaining excellent distance acuity [11]. The RayOne EMV (RAO200E, Rayner) employs a hydrophilic acrylic platform with induced positive spherical aberration to achieve a similar effect with minimal dysphotopsia [9]. Clinical studies and meta-analyses have shown that enhanced monofocal IOLs improve uncorrected intermediate vision compared with standard monofocal lenses while preserving distance visual acuity and inducing fewer photic phenomena than multifocal designs [11].

Despite these advantages, refractive evaluation of enhanced monofocal IOLs presents unique challenges. Subjective manifest refraction remains the clinical gold standard, relying on patient responses to define optimal correction [12]. Automated refraction, particularly wavefront-based aberrometry, provides objective measurements by analyzing the propagation of light through the optical system. Devices such as the iTrace (ray-tracing) and LadarWave (Hartmann–Shack) offer detailed aberrometric assessment; however, their agreement with subjective refraction may be influenced by IOL designs that intentionally modify spherical aberration and higher-order aberration profiles [13,14,15].

Importantly, a correlation between automated and subjective refraction does not necessarily indicate agreement between the methods, as two techniques may show strong correlation while exhibiting systematic bias or wide clinically relevant limits of agreement [16]. This distinction is particularly important in eyes implanted with enhanced monofocal IOLs, where refractive values are often close to emmetropia and small absolute differences may be magnified when expressed as relative metrics [16,17].

Previous studies have suggested that automated refraction may overestimate myopia or astigmatism in pseudophakic eyes, especially in the presence of higher-order aberrations or aberration-modifying optics [16,17]. In this context, agreement-based analyses, such as Bland–Altman plots, are considered the standard approach for comparing refractive measurement techniques, as they allow for direct visualization of systematic bias and variability across the measurement range rather than relying solely on association metrics [18].

Accurate postoperative refraction is essential for spectacle independence and underpins patient satisfaction and postoperative refractive planning [18,19]. However, data comparing subjective manifest refraction with wavefront-based automated refraction in eyes implanted with enhanced monofocal IOLs remain limited, with most existing literature focusing on multifocal or diffractive IOL designs [20,21]. Moreover, few studies have applied agreement-based statistical methods or accounted for repeated measures and bilateral eyes when evaluating refractive outcomes in this setting, representing an important gap in the literature.

Accordingly, the present study was designed to compare subjective manifest refraction with wavefront-based automated refraction using iTrace and LadarWave in eyes implanted with two enhanced monofocal IOLs (DIB00 and RAO200E) and a standard aspheric monofocal IOL (ZCB00). The primary objective was to assess agreement between methods using clinically meaningful metrics, including mean bias, limits of agreement, and absolute error thresholds, rather than relying solely on correlation-based measures. Secondary objectives included evaluating refractive variability across IOL designs and exploring how differences in optical design may be associated with discrepancies in automated refractive measurements, without assuming a direct causal relationship.

## 2. Methods

### 2.1. Study Design and Ethics

This was a retrospective cohort study including eyes that underwent cataract surgery with implantation of one of three monofocal intraocular lenses between March 2021 and December 2022 at the Storm Eye Institute, Medical University of South Carolina. This study adhered to the tenets of the Declaration of Helsinki and was approved by the Institutional Review Board of the Medical University of South Carolina.

Given the retrospective nature of this study and the use of anonymized data, informed consent was waived.

Inclusion criteria required uncomplicated cataract surgery and a postoperative corrected distance visual acuity (CDVA) of 20/40 or better at follow-up, to ensure reliable subjective refraction.

Exclusion criteria included strabismus, ocular comorbidities affecting capsular bag or zonular stability (such as pseudoexfoliation syndrome or prior ocular trauma), clinically evident intraocular lens tilt or decentration, poor fixation, or any corneal or retinal pathology that could compromise refractive measurements.

In bilateral cases, both eyes were included only when the same IOL model was implanted in both eyes.

### 2.2. Intraocular Lenses

Three intraocular lenses were analyzed. The Tecnis Eyhance (DIB00, Johnson & Johnson Vision, New Brunswick, NJ, USA) is a hydrophobic acrylic, enhanced monofocal, with a continuous aspheric anterior surface, designed to induce positive spherical aberration and thereby extend the depth of focus. The RayOne EMV (RAO200E, Rayner, Worthing, UK) is a hydrophilic acrylic enhanced monofocal that also employs controlled positive spherical aberration to improve intermediate vision while preserving distance acuity. The Tecnis ZCB00 (Johnson & Johnson Vision, New Brunswick, NJ, USA) is a hydrophobic acrylic aspheric monofocal lens with negative spherical aberration, designed to compensate for average corneal spherical aberration, optimized for distance vision, and was used as the control group.

In all cases, IOL power was calculated with the IOLMaster 700 (Carl Zeiss Meditec AG, Jena, Germany) using the Barrett Universal II formula, aiming for emmetropia.

### 2.3. Surgical Technique

All surgeries were performed by a single experienced surgeon (K.M.R.) under topical anesthesia. Phacoemulsification was carried out with the Centurion Vision System (Alcon, Fort Worth, TX, USA) through a 2.2 mm temporal clear corneal incision. A continuous curvilinear capsulorhexis of approximately 5.5 mm was created, followed by hydrodissection, nuclear emulsification, cortical aspiration, and in-the-bag IOL implantation using a preloaded injector. No intraoperative complications were recorded. Postoperatively, patients received topical moxifloxacin 0.5% for one week, and prednisolone acetate 1% tapered over 4 weeks.

### 2.4. Preoperative and Postoperative Assessment

Preoperative evaluation included uncorrected distance visual acuity (UCDVA), corrected distance visual acuity (CDVA), slit-lamp biomicroscopy, dilated fundus examination, and optical biometry. Postoperative assessments were performed between one and three months after surgery, when refractive stability is typically achieved.

Manifest, or subjective, refraction was performed by masked examiners using a phoropter under mesopic illumination, with the maximum plus lens for the maximum acuity endpoint.

Automated refraction was obtained with two aberrometers: the iTrace (Tracey Technologies, Houston, TX, USA), which uses ray-tracing technology to project laser beams across the pupil and reconstruct the ocular wavefront, and the LadarWave (Alcon, Fort Worth, TX, USA), a Hartmann–Shack device using microlens arrays and Zernike polynomial reconstruction.

For each automated device, three consecutive measurements were obtained per eye under mesopic lighting conditions, and the mean of consistent measurements was used for analysis. Measurements were accepted only when device-specific quality criteria were met (quality score > 7/10 for iTrace and signal quality > 80% for LadarWave).

Manifest refraction was performed first, followed by automated measurements. Due to the retrospective design, the order of the automated devices was not standardized.

Pupil diameter at the time of automated refraction was recorded from device outputs; however, pupil size was not standardized across measurements. This was acknowledged as a potential source of variability, particularly given the pupil-dependent effects of spherical aberration in enhanced monofocal IOL designs.

### 2.5. Definitions and Outcome Measures

The main refractive outcomes were sphere, cylinder, and spherical equivalent, defined as the sphere plus half the cylinder.

Astigmatism was additionally analyzed using power vector components (J_0_ and J_45_), derived from the cylinder magnitude and axis to account for the vectorial nature of refractive astigmatism.

To quantify discrepancies between automated and manifest refraction, the percentage variation (PV) was calculated. PV was defined as the difference between the automated and the subjective value divided by the absolute value of the subjective refraction and multiplied by 100.

Because manifest refraction values in eyes targeted for emmetropia are often close to zero, even small absolute differences can generate disproportionately high PV values. Therefore, PV was interpreted only as a relative index of variability and was never considered in isolation or as a primary agreement metric.

Primary agreement analyses focused on mean signed difference (bias), mean absolute error (MAE), root mean square error (RMSE), 95% limits of agreement (Bland–Altman), and the proportion of eyes within clinically relevant thresholds (±0.25 D, ±0.50 D, and ±1.00 D).

Raw data were reanalyzed from the original Excel files, and when minor discrepancies were observed with previous tables, recalculations from the raw data were prioritized to ensure accuracy.

### 2.6. Statistical Analysis

Data distribution for spherical equivalent (SE) was assessed using the Shapiro–Wilk test within each IOL group and was approximately normal; therefore, the results are presented as mean ± standard deviation.

To compare spherical equivalent (SE) across IOL types and measurement methods while accounting for correlated observations, we fitted a linear mixed-effect model. Method was specified as a three-level categorical factor (Manifest, iTrace, LadarWave), IOL type as a three-level factor (DIB00, RAO200E, ZCB00), and an IOL×method interaction was included.

A random intercept for the patient was used to account for the clustering of bilateral eyes within subjects. Manifest was defined as the reference level for Method, and DIB00 as the reference level for IOL type; therefore, fixed-effect estimates for iTrace and LadarWave represent mean SE differences relative to Manifest, and estimates for RAO200E and ZCB00 represent mean differences relative to DIB00. The interaction term was evaluated using a likelihood-ratio test comparing models with and without the interaction (maximum-likelihood fits).

Agreement between manifest and automated methods was further characterized using Bland–Altman analysis (mean bias and 95% limits of agreement), Pearson correlation, Lin’s concordance correlation coefficient (CCC), and intraclass correlation coefficients (ICC; two-way mixed-effect, absolute agreement, single measurements) with 95% confidence intervals.

Sensitivity analyses using OD-only and OS-only datasets were performed to confirm robustness.

Statistical significance was set at *p* < 0.05. This approach replaced independent-sample and one-way ANOVA analyses used in preliminary evaluations.

## 3. Results

### 3.1. Demographics and Visual Acuity

A total of 84 eyes from 42 patients were analyzed, with 22 eyes implanted with the Tecnis Eyhance (DIB00), 31 with the RayOne EMV (RAO200E), and 31 with the Tecnis ZCB00. The mean age of the cohort was 68.2 ± 10.2 years, with no statistically significant difference across groups (*p* = 0.426). Gender distribution showed a higher proportion of female patients in the DIB00 (86.4%) and RAO200E (67.7%) groups than in the ZCB00 group (51.6%), with statistical significance (*p* = 0.030). Visual acuity outcomes were excellent and comparable among the three groups. Mean UCDVA was 0.09 ± 0.07 logMAR in the DIB00 group, 0.10 ± 0.12 logMAR in the RAO200E group, and 0.07 ± 0.14 logMAR in the ZCB00 group (*p* = 0.719). CDVA was similarly good, with values of 0.01 ± 0.02 logMAR, 0.02 ± 0.07 logMAR, and 0.01 ± 0.03 logMAR for the DIB00, RAO200E, and ZCB00 groups, respectively (*p* = 0.409) (Table 1).

All included eyes met the predefined inclusion criterion of postoperative CDVA ≥ 20/40, supporting the reliability of subjective refraction.

### 3.2. Subjective Manifest Refraction

Manifest refraction revealed no statistically significant differences in spherical power among the three intraocular lenses, with mean values of −0.29 ± 0.45 D for DIB00, −0.23 ± 0.53 D for RAO200E, and −0.41 ± 0.47 D for ZCB00 (*p* = 0.177).

In contrast, cylindrical refraction differed significantly across groups (*p* = 0.006). Mean cylinder was −0.26 ± 0.24 D in the DIB00 group, −0.17 ± 0.28 D in the RAO200E group, and −0.41 ± 0.38 D in the ZCB00 group. Post hoc analysis confirmed a statistically significant difference between RAO200E and ZCB00 (*p* = 0.021), with the ZCB00 group demonstrating higher residual astigmatism.

The spherical equivalent (SE) also showed a statistically significant intergroup difference (*p* = 0.027). Mean SE values were −0.03 ± 0.41 D for DIB00, −0.20 ± 0.32 D for RAO200E, and −0.08 ± 0.44 D for ZCB00. RAO200E demonstrated a more myopic postoperative spherical equivalent compared with ZCB00 (*p* = 0.035).

**Figure 1:**
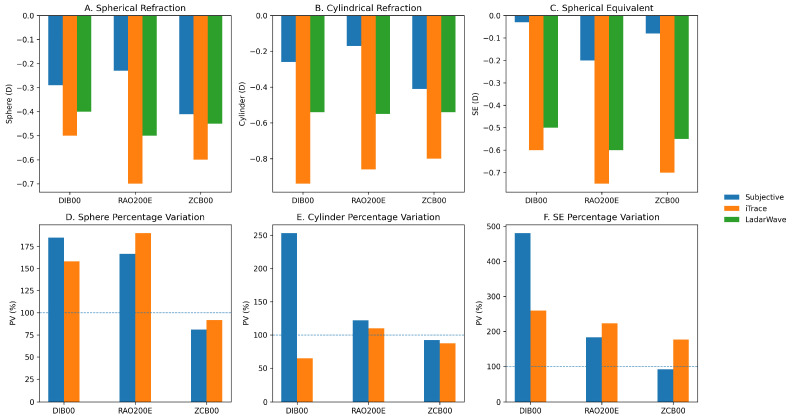
Comparison of subjective and automated refraction outcomes across three monofocal intraocular lenses (DIB00, RAO200E, ZCB00). (**A**) Spherical refraction by lens and device. (**B**) Cylindrical refraction by lens and device. (**C**) Spherical equivalent (SE) by lens and device. (**D**) Sphere percentage variation by device and lens. (**E**) Cylinder percentage variation by device and lens. (**F**) SE percentage variation by device and lens. Subjective manifest refraction is shown in grey, iTrace (ray-tracing aberrometer) in blue, and LadarWave (Hartmann–Shack aberrometer) in orange. Bars represent mean values for each IOL group. Panels (**A**–**C**) display absolute dioptric values (**D**), with the zero line indicated. Panels (**D**–**F**) display percentage variation (PV, %), with the dashed line indicating the 100% reference. Percentage variation was defined as the difference between automated and subjective refraction, expressed as a percentage of the absolute subjective value. Because manifest values were frequently close to emmetropia, percentage variation values may appear numerically high and should be interpreted together with absolute differences. Statistical significance was assessed using one-way ANOVA with Tukey’s post hoc test (*p* < 0.05).

Subjective manifest refraction outcomes are detailed in Table 1 and illustrated in Figure 1A–C.

### 3.3. Automated Refraction

Automated refraction demonstrated greater variability and systematic differences compared with subjective manifest refraction, particularly in eyes implanted with enhanced monofocal intraocular lenses.

In the DIB00 group, the mean subjective cylinder was −0.26 ± 0.24 D, compared with −0.94 ± 0.64 D measured with iTrace and −0.54 ± 0.35 D measured with LadarWave (*p* < 0.001). Similarly, in the RAO200E group, subjective cylinder was −0.17 ± 0.28 D, whereas automated measurements yielded −0.86 ± 0.58 D with iTrace and −0.55 ± 0.30 D with LadarWave (*p* < 0.001). In the ZCB00 group, subjective cylinder was −0.41 ± 0.38 D, compared with −0.80 ± 0.42 D with iTrace and −0.54 ± 0.36 D with LadarWave (*p* = 0.001).

When comparing automated devices across IOL groups, no statistically significant inter-lens differences were observed for LadarWave cylinder measurements (*p* = 0.958). In contrast, iTrace demonstrated consistently higher cylindrical values, particularly in the enhanced monofocal groups (Figure 1B).

For SE, agreement analysis revealed a systematic negative bias between automated refraction and subjective manifest refraction, indicating a more myopic automated estimate. In the DIB00 group, the mean bias (automated − manifest) was −0.26 D with iTrace and −0.54 D with LadarWave. In the RAO200E group, corresponding biases were −0.51 D and −0.73 D, respectively. In contrast, the ZCB00 group demonstrated smaller biases, particularly with iTrace (−0.05 D).

Agreement metrics, including mean absolute error (MAE), root mean square error (RMSE), 95% limits of agreement (LoA), and the proportion of eyes within ±0.50 D, are summarized in Table 2**.** The proportion of eyes within ±0.50 D for SE was markedly lower in enhanced monofocal IOLs (24–57%) than in the ZCB00 group (65–94%), indicating better agreement with the standard aspheric monofocal lens.

**Figure 2:**
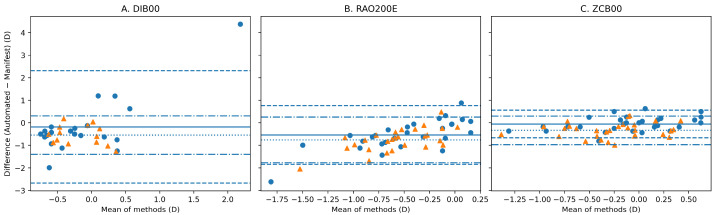
Bland–Altman agreement analysis for spherical equivalent (SE). Bland–Altman plots comparing automated refraction with subjective manifest refraction for spherical equivalent (SE) in eyes implanted with (**A**) Tecnis Eyhance (DIB00), (**B**) RayOne EMV (RAO200E), and (**C**) Tecnis ZCB00 intraocular lenses. Circles represent iTrace and triangles represent LadarWave measurements. For each device within each IOL group, the solid horizontal line indicates the mean signed difference (bias; automated − manifest), and the dashed lines indicate the 95% limits of agreement (bias ± 1.96 SD).

**Figure 3:**
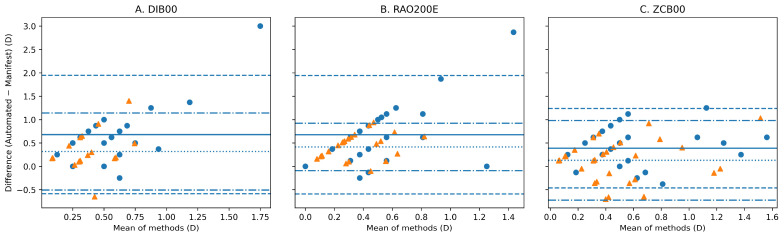
Bland–Altman agreement analysis for cylinder magnitude. Bland–Altman plots comparing automated refraction (iTrace and LadarWave) with subjective manifest refraction for cylinder magnitude (absolute value) in eyes implanted with (**A**) Tecnis Eyhance (DIB00), (**B**) RayOne EMV (RAO200E), and (**C**) Tecnis ZCB00 intraocular lenses. Circles represent iTrace and triangles represent LadarWave measurements. Solid horizontal lines indicate the mean signed difference (bias; automated − manifest), and dashed lines indicate the 95% limits of agreement (bias ± 1.96 SD). The cylinder was analyzed as a magnitude because axis data were unavailable for all eyes.

Bland–Altman agreement analysis for spherical equivalent is illustrated in Figure 2, showing wider limits of agreement for DIB00 and RAO200E than for ZCB00. Bland–Altman plots for cylinder magnitude are shown in Figure 3, further highlighting greater dispersion and reduced agreement in enhanced monofocal IOLs.

### 3.4. Percentage Variation

The percentage variation (PV) analysis further illustrated the discrepancies between automated and subjective refraction methods; however, this metric was interpreted with caution and considered a secondary, supportive analysis rather than a primary measure of agreement.

For cylindrical refraction, mean PV values using iTrace reached 253.2% in the DIB00 group, 121.8% in the RAO200E group, and 92.2% in the ZCB00 group (*p* = 0.016). Using LadarWave, the corresponding PV values were 64.8%, 109.8%, and 87.3%, respectively (*p* = 0.166). For spherical power, iTrace showed mean PV values of 184.8% in DIB00, 166.6% in RAO200E, and 80.9% in ZCB00 (*p* = 0.205), while LadarWave showed 157.9%, 189.9%, and 91.7%, respectively (*p* = 0.018).

For spherical equivalent (SE), PV values were particularly elevated, reaching 480.8% for DIB00 with iTrace, 182.5% for RAO200E with iTrace, and 92.0% for ZCB00 with iTrace (*p* = 0.001). With LadarWave, the corresponding values were 259.2%, 222.9%, and 176.5%, respectively (*p* = 0.072) (Figure 1D–F).

These large percentage values predominantly reflect the mathematical instability of PV when subjective refraction values are close to emmetropia, rather than large absolute refractive errors. As such, PV was not used to infer clinical agreement or interchangeability between methods; instead, it served as a relative indicator highlighting variability patterns across IOL designs.

Accordingly, PV findings were interpreted in conjunction with absolute dioptric differences, Bland–Altman limits of agreement, clinically relevant thresholds, and mixed-effect modeling, as summarized in Table 2 and Figure 2 and Figure 3.

### 3.5. Correlation and Agreement

Analysis of the relationship between subjective and automated refraction revealed important limitations of agreement, particularly in eyes implanted with enhanced monofocal intraocular lenses. Correlation analyses were therefore interpreted cautiously, recognizing that correlation reflects association rather than agreement between measurement methods.

Pearson correlation coefficients were weak in the DIB00 and RAO200E groups across most refractive parameters (r < 0.35, *p* > 0.05), indicating poor linear association between automated and subjective measurements. In contrast, the ZCB00 group demonstrated moderate correlations for spherical equivalent measured with iTrace (r = 0.46, *p* = 0.01) and for cylinder magnitude (r = 0.41, *p* = 0.02), reflecting a narrower range of differences in the standard aspheric monofocal lens group.

Intraclass correlation coefficients (ICC) were calculated using a two-way mixed-effect model for absolute agreement and single measurements, with 95% confidence intervals. ICC values were uniformly low in the enhanced monofocal groups (ICC < 0.12), confirming poor agreement despite occasional moderate correlations. In the ZCB00 group, ICC values were higher. Still, they remained below thresholds typically considered indicative of strong agreement, reinforcing that automated and subjective refraction methods are not interchangeable even in conventional monofocal IOLs.

To further distinguish agreement from correlation, Lin’s concordance correlation coefficient (CCC) was additionally evaluated. CCC values mirrored the ICC findings, demonstrating lower concordance in enhanced monofocal IOLs and substantially higher concordance in the ZCB00 group, consistent with the narrower Bland–Altman limits of agreement observed in Figure 2 and Figure 3.

Overall, these findings emphasize that correlation metrics alone may be misleading when assessing agreement between refraction methods, and that absolute differences, Bland–Altman analyses, concordance metrics, and clinically relevant thresholds provide a more accurate characterization of measurement performance across IOL designs.

### 3.6. Linear Mixed-Effect Model Analysis

A linear mixed-effect model was fitted with refraction method specified as a three-level categorical factor (Manifest, iTrace, LadarWave), IOL type (DIB00, RAO200E, ZCB00), and the IOL×method interaction as fixed effects, with a random intercept for patient. The IOL×method interaction was statistically significant (likelihood-ratio test, *p* = 0.013), indicating that the magnitude of automated–manifest SE differences varied by IOL design. In stratified contrasts (Table 3), automated methods showed a consistent myopic bias versus manifest refraction for enhanced monofocal IOLs: DIB00 (iTrace −0.269 D, *p* = 0.0003; LadarWave −0.555 D, *p* < 0.001) and RAO200E (iTrace −0.552 D, *p* < 0.001; LadarWave −0.785 D, *p* < 0.001). In the standard aspheric monofocal ZCB00 group, iTrace did not differ significantly from manifest refraction (−0.054 D, *p* = 0.616), whereas LadarWave retained a significant myopic bias (−0.338 D, *p* = 0.0016).

**Figure 4:**
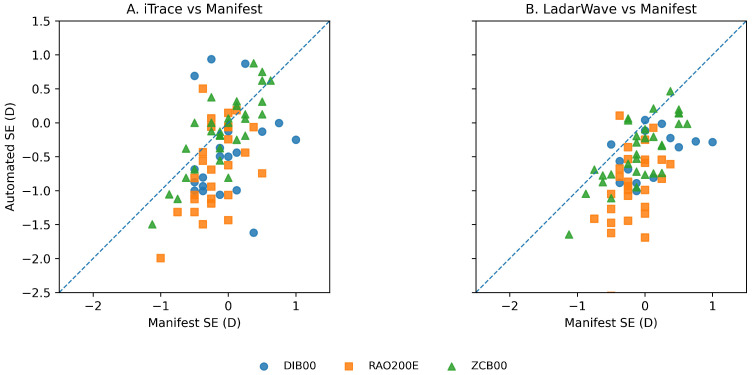
Scatterplots showing agreement between automated and subjective manifest spherical equivalent (SE). (**A**) iTrace versus manifest refraction. (**B**) LadarWave versus manifest refraction. Each point represents one eye, color-coded by intraocular lens (IOL) type: Tecnis Eyhance (DIB00), RayOne EMV (RAO200E), and Tecnis ZCB00. The dashed line represents the line of identity (y = x). Enhanced monofocal IOLs (DIB00 and RAO200E) show greater dispersion around the identity line, indicating lower agreement, whereas the standard aspheric monofocal IOL (ZCB00) demonstrates tighter clustering and better deals with subjective refraction.

Vector astigmatism agreement (J0 and J45) further supported the cylinder findings, showing lower errors for the standard aspheric monofocal ZCB00 compared with the enhanced monofocal designs. J0 mean absolute error ranged from 0.30–0.36 D in DIB00/RAO200E versus 0.22–0.25 D in ZCB00, and J45 mean absolute error ranged from 0.20–0.24 D in DIB00/RAO200E versus 0.14–0.16 D in ZCB00 (Table 4). Consistent with these agreement metrics, scatterplots of spherical equivalent showed greater dispersion from the line of identity for DIB00 and RAO200E than for ZCB00, indicating reduced agreement between automated and manifest measurements in enhanced monofocal IOLs (Figure 4).

## 4. Discussion

This study compared subjective manifest refraction with wavefront-based automated refraction using iTrace and LadarWave in eyes implanted with two enhanced monofocal intraocular lenses (DIB00 and RAO200E) and a standard aspheric monofocal IOL (ZCB00). By incorporating agreement-based metrics, Bland–Altman analysis, vector astigmatism assessment, and linear mixed-effect modeling to account for bilateral eyes, the present study provides a more robust and methodologically rigorous evaluation of refractive performance across IOL designs than prior reports. The main findings were that enhanced monofocal IOLs exhibited greater variability and poorer agreement between automated and subjective refraction than the standard aspheric monofocal lens, particularly for cylinder and spherical equivalent, and that these discrepancies persisted even after accounting for within-patient correlation and repeated measurements, with evidence that the automated–manifest SE differences varied by IOL design (method × IOL interaction).

The ZCB00 demonstrated more consistent agreement between subjective and automated refraction, in line with its optical design, which incorporates negative spherical aberration to compensate for average corneal spherical aberration and optimize distance vision [2,3]. In contrast, both DIB00 and RAO200E intentionally induce positive spherical aberration to extend the depth of focus [9,10,11]. This optical modification was associated with wider Bland–Altman limits of agreement, higher mean absolute errors, and a lower proportion of eyes within clinically acceptable thresholds (±0.50 D) for automated refraction, suggesting reduced interchangeability between automated and subjective methods in enhanced designs. These findings are consistent with previous reports indicating that automated and wavefront-based refraction may overestimate refractive error in eyes with aberration-modifying optics [20,21,22,23].

Although subjective SE was close to emmetropia in all groups, RAO200E demonstrated a slightly more myopic mean SE than ZCB00 (−0.20 ± 0.32 D vs. −0.08 ± 0.44 D, *p* = 0.035). This intergroup difference was small in magnitude and below commonly accepted thresholds for clinical relevance, underscoring the importance of distinguishing statistical significance from clinical significance when interpreting postoperative refractive outcomes.

A device-dependent difference was observed, and the mixed-effect model supported that the magnitude of automated–manifest SE differences varied by IOL design (method × IOL interaction). In the mixed-effect contrasts, the myopic bias relative to manifest refraction was larger in the enhanced monofocal IOLs. In contrast, in the ZCB00 group, iTrace did not differ significantly from manifest refraction, while LadarWave retained a significant myopic bias. The iTrace showed larger deviations from manifest refraction, particularly in enhanced monofocal IOLs, suggesting greater sensitivity to higher-order aberrations and optical scatter. By contrast, LadarWave (Hartmann–Shack) yielded values closer to subjective refraction in some eyes, although variability persisted. These findings likely reflect fundamental differences in wavefront sampling and reconstruction between ray-tracing and Hartmann–Shack systems. They are consistent with prior reports of method-dependent discrepancies in eyes implanted with aberration-modifying IOLs [23,24,25,26,27,28,29,30].

Astigmatism analysis further reinforced these observations. Automated measurements tended to overestimate cylinder magnitude in enhanced monofocal IOLs, with a substantial proportion of eyes exceeding ±0.50 D when compared with subjective refraction. To address the vectorial nature of astigmatism and to address the limitations of scalar cylinder analysis, power vector analysis (J_0_ and J_45_) was performed. Vector analysis confirmed greater dispersion and reduced agreement in enhanced monofocal designs, indicating that discrepancies extended beyond magnitude alone and included directional components of astigmatism.

From a clinical perspective, these results emphasize that subjective manifest refraction remains the gold standard for postoperative assessment in eyes implanted with premium or aberration-modifying IOLs. Automated aberrometry can support clinical decision-making, but its outputs should be interpreted as complementary rather than interchangeable with subjective refraction. For example, a patient implanted with an Eyhance lens may present with an automated cylinder near −1.00 D on iTrace, while manifest refraction reveals only −0.25 D; reliance on automated data alone could therefore lead to overcorrection or unnecessary optical enhancement. Careful integration of subjective refraction, visual acuity, and aberrometric information is essential to avoid misinterpretation.

Several limitations of this study should be acknowledged. The retrospective design and relatively small sample size may limit generalizability, and the imbalance in gender distribution across groups could reflect selection bias. Although linear mixed-effect models were used to account for bilateral eyes and repeated measures, residual confounding cannot be fully excluded. Pupil diameter was recorded but not standardized, and measurements were obtained under mesopic conditions, which may have influenced wavefront-derived refraction given the pupil-dependent effects of spherical aberration in enhanced monofocal IOL designs. Functional outcomes such as contrast sensitivity or defocus curves were not assessed and could provide additional insight into visual performance in future investigations.

Mechanistic interpretations should also be made with caution. Although induced positive spherical aberration is a plausible explanation for the observed discrepancies, higher-order aberrations were not directly correlated with refractive differences in this dataset. Accordingly, the relationship between IOL-induced aberrations and automated refraction variability should be regarded as hypothesis-generating rather than causal, and future studies incorporating pupil-normalized aberrometry and direct HOA correlation are warranted.

In conclusion, enhanced monofocal intraocular lenses (DIB00 and RAO200E) exhibit greater refractive variability and poorer agreement between automated wavefront-based refraction and subjective manifest refraction compared with the standard aspheric monofocal ZCB00, reflecting differences in optical design. While wavefront aberrometers provide valuable objective information, their measurements should be interpreted with caution in eyes implanted with enhanced monofocal IOLs. Subjective manifest refraction remains indispensable for accurate postoperative evaluation, and future studies should focus on pupil-normalized analyses, direct assessment of higher-order aberrations, and refinement of automated algorithms to better accommodate aberration-modifying IOL designs.

## Figures and Tables

**Table 1:** Demographics, visual acuity, and postoperative subjective refraction by IOL group.

Parameter	DIB00 (*n* = 22 Eyes)	RAO200E (*n* = 31 Eyes)	ZCB00 (*n* = 31 Eyes)	*p*-Value
Age (years)	67.9 ± 9.8	69.0 ± 11.1	67.8 ± 9.7	0.426
Female (%)	86.4	67.7	51.6	0.030 *
UCDVA (logMAR)	0.09 ± 0.07	0.10 ± 0.12	0.07 ± 0.14	0.719
CDVA (logMAR)	0.01 ± 0.02	0.02 ± 0.07	0.01 ± 0.03	0.409
Sphere (D)	−0.29 ± 0.45	−0.23 ± 0.53	−0.41 ± 0.47	0.177
Cylinder (D)	−0.26 ± 0.24	−0.17 ± 0.28	−0.41 ± 0.38	0.006 *
Spherical equivalent (D)	−0.03 ± 0.41	−0.20 ± 0.32	−0.08 ± 0.44	0.027 *

Data are presented as mean ± SD unless otherwise indicated. Statistically significant at *p* < 0.05 (*).

**Table 2:** Agreement between automated and subjective manifest spherical equivalent (SE).

IOL	Device	Bias (D)	MAE (D)	RMSE (D)	95% LoA (D)	±0.50 D (%)
DIB00	iTrace	−0.26	0.56	0.84	−1.82 to 1.31	57
DIB00	LadarWave	−0.54	0.58	0.71	−1.43 to 0.36	38
RAO200E	iTrace	−0.51	0.64	0.83	−1.79 to 0.78	38
RAO200E	LadarWave	−0.73	0.78	0.89	−1.75 to 0.30	24
ZCB00	iTrace	−0.05	0.25	0.31	−0.66 to 0.55	94
ZCB00	LadarWave	−0.34	0.39	0.47	−0.96 to 0.29	65

Bias = automated − subjective. LoA = limits of agreement (Bland–Altman). ±0.50 D indicates the proportion of eyes within a clinically acceptable threshold.

**Table 3:** Mixed-effect model contrasts for spherical equivalent (SE): automated methods versus manifest refraction, stratified by IOL type.

IOL	Contrast (Automated − Manifest)	Estimate (D)	95% CI (D)	*p*-Value
DIB00	iTrace vs. Manifest	−0.269	−0.414 to −0.123	0.0003
DIB00	LadarWave vs. Manifest	−0.555	−0.706 to −0.403	<0.001
RAO200E	iTrace vs. Manifest	−0.552	−0.765 to −0.339	<0.001
RAO200E	LadarWave vs. Manifest	−0.785	−1.006 to −0.564	<0.001
ZCB00	iTrace vs. Manifest	−0.054	−0.264 to 0.157	0.616
ZCB00	LadarWave vs. Manifest	−0.338	−0.549 to −0.128	0.0016

Notes/footnote: Linear mixed-effect model with fixed effects for IOL type, method (Manifest as the reference; iTrace and LadarWave), and the IOL×method interaction; random intercept for patient. Estimates (D) represent mean differences (Automated − Manifest); negative values indicate a more myopic SE by the automated method.

**Table 4:** Vector astigmatism agreement between automated and subjective refraction.

IOL	Device	J0 Bias (D)	J0 MAE (D)	J45 Bias (D)	J45 MAE (D)
DIB00	iTrace	−0.17	0.30	−0.02	0.20
RAO200E	iTrace	−0.13	0.32	0.04	0.21
ZCB00	iTrace	−0.18	0.25	0.01	0.16
DIB00	LadarWave	−0.21	0.34	−0.03	0.23
RAO200E	LadarWave	−0.19	0.36	0.05	0.24
ZCB00	LadarWave	−0.09	0.22	0.02	0.14

Astigmatism was converted to power vectors (J0, J45). Lower values indicate better agreement.

## Data Availability

The original contributions presented in this study are included in this article. Further inquiries can be directed to the corresponding author.
